# Asymptomatic Bacteriuria among Pregnant Women Attending Antenatal Care at Hiwot Fana Specialized University Hospital, Harar, Eastern Ethiopia: Magnitude, Associated Factors, and Antimicrobial Susceptibility Pattern

**DOI:** 10.1155/2020/1763931

**Published:** 2020-07-20

**Authors:** Mekuria Edae, Zelalem Teklemariam, Fitsum Weldegebreal, Degu Abate

**Affiliations:** ^1^Hiwot Fana Specialized University Hospital, College of Health and Medical Sciences, Haramaya University, P.O. Box 235, Harar, Ethiopia; ^2^Department of Medical Laboratory Sciences, College of Health and Medical Sciences, Haramaya University, P.O. Box 235, Harar, Ethiopia

## Abstract

**Background:**

Asymptomatic bacteriuria is one of the major risk factors for the development of urinary tract infections during pregnancy which accounts for about 70% of the cases. However, there is no guideline which recommends routine screening of pregnant women for asymptomatic bacteriuria in most of developing countries including Ethiopia. Thus, the aim of this study was to determine the magnitude, associated factors, and antimicrobial susceptibility pattern of asymptomatic bacteriuria among pregnant women.

**Methods:**

A cross-sectional study was conducted from March to April 2019. Data were collected through face-to-face interview and analyzed using Statistical Package for Social Science version 22. A test of association was performed using logistic regression and *P* value less than 0.05 was considered statistically significant.

**Results:**

The overall prevalence of asymptomatic bacteriuria was 19.9%. Direction of wiping after genital wash, postcoital urination, and catheterization were factors significantly associated with asymptomatic bacteriuria. Most of the isolated Gram positive were highly sensitive to Ceftriaxone (90.9%). Coagulase-negative staphylococci showed higher sensitivity to Augmentin (75.0) and Ceftriaxone (87.5%), whereas they showed resistance to Clindamycin (68.7%) and Ampicillin (62.5%). Gram-negative bacteria isolates showed higher sensitivity to Ceftriaxone (88.2%), Gentamycin (67.5%), and Augmentin (64.7%), while they showed resistance to Ampicillin (70.5%) and Clindamycin (50.0%).

**Conclusion:**

The overall prevalence of asymptomatic bacteriuria among pregnant women in this study was high. Direction of wiping after genital wash, catheterization, and postcoital urination increases the odds of asymptomatic bacteriuria. Therefore, health education on the predisposing factors is strongly recommended.

## 1. Background

Asymptomatic bacteriuria (ASB) is a condition in which urine culture reveals a significant growth of bacteria which is greater than or equal to 10^5^ colony-forming unit per milliliter (ml) of urine taken from a clean catch midstream urine but without the patient showing symptoms such as genitourinary tract infection [1]. Pregnant women are two times more commonly affected than age-matched non-pregnant women. Asymptomatic bacteriuria can be symptomatic and asymptomatic urinary tract infection [1, 2].

Early screening and treating promptly of ASB during pregnancy can prevent further complication of bacteriuria in pregnant women [3]. According to the American College of Obstetricians and Gynecologists, screening of ASB is recommended in all pregnant women [2]. However, in many hospitals in developing countries including Ethiopia, routine urine culture test is not carried out for antenatal women probably due to cost implication and time factor for culture result (usually 48 hours period) [4].

Asymptomatic bacteriuria is one of the major risk factors for the development of UTIs during pregnancy which accounts for about 70% of the cases [5]. Globally, asymptomatic bacteriuria affects 2–10% of all pregnant women [6]. But it was reported at different level in different parts of the world.

The burden of this asymptomatic bacteriuria among pregnant women in Ethiopia also varies with the location of study [5, 7–10]. In Ethiopia, there is no guideline that recommends screening of ASB for the pregnant women regardless of the symptoms and sign. If untreated at the time of ASB, it causes about 40% of cystitis (infection of bladder) and 30% pyelonephritis (infection of kidney) [11]. It also affects the infants by causing premature delivery, low birth weight, intrauterine growth retardation, preterm labor, intrauterine fetal death, and in general, it increased prenatal mortality and morbidity [12]. It is estimated that 30–50% of pregnant women without on-time treatment of ASB in pregnancy will progress to symptomatic urinary tract infection. Therefore, appropriate screening and treatment of ASB will reduce the infection rate to 3% [4].

There are number of studies conducted in different part of the world about magnitude of asymptomatic bacteriuria, antimicrobials susceptibility and its associated factors for asymptomatic bacteriuria among pregnant women [1, 13]. However, to our information, there is no study conducted in Harar town. Therefore, this study was used to determine the magnitude of ASB, the drug susceptibility pattern, and associated factors of asymptomatic bacteriuria among pregnant women attending antenatal care (ANC) at Hiwot Fana Specialized University Hospital.

## 2. Methods and Materials

### 2.1. Study Area, Design, and Period

An institutional based cross-sectional study was conducted on pregnant women attending antenatal care at Hiwot Fana Specialized University Hospital from March to April 2019. Hiwot Fana Specialized University Hospital is in Harari People's National Regional State, Harar, Eastern Ethiopia. Currently, the hospital serves to about 5.2 million communities around Harar and neighboring regions such as, Oromia Regional State, Dire Dawa Administrative Council, and Ethiopia Somali Regional State.

### 2.2. Sample Size Determination and Sampling Technique

The sample size was determined using a single population formula taking a prevalence of ASB (21.2%) from the study conducted in Adigrat General Hospital [5] with the degree of precision set at 0.05 and at 95% confidence interval. The final sample size was 281. Study participants were selected by a convenient sampling technique.

### 2.3. Data Collection Methods

#### 2.3.1. Data Collection Instruments

Data were collecetd using structured questionnaires developed from different literature [5, 9, 13]. The questionnaire contains two parts: sociodemographic characteristics and clinical and pregnancy-related factors. Laboratory examination of urine for the isolation of bacteria causing ASB and their antimicrobial susceptibility pattern was used to collect laboratorybased data.

#### 2.3.2. Data Collection Procedure

First, all pregnant women who came for ANC services were screened whether they have signs and symptoms of UTI such as burning sensation, suprapubic pain, frequent urination, and fever, and then data were collected by the senior nurse from pregnant mothers who have no signs and symptoms of UTI. Other information such as history of hypertension, history of diabetes, and hemoglobin level were obtained from the medical record book of the pregnant women. Questionnairebased data were collected by face to face interview using pretested structured questionnaires.

The study participants were instructed how to collect 10–15 mL of standard midstream urine by the “clean catch” method. They were instructed to wash their hands, cleanse the genitals with clean water, dry the area with a sterile gauze pad, and collect the middle urine into a wide-mouthed screw-capped universal urine container with labia held apart. Then, study participants identification number, date, and time of collection were labelled on the outside of the container. The collected sample was kept in a cold box and sent to Haramaya University College of Health and Medical Sciences Department of Medical Laboratory Sciences within 30 minutes of collection time.

#### 2.3.3. Urine Culture and Identification of Bacteria

The aseptically collected and well-mixed urine sample was inoculated onto CLED media, blood agar, and MacConkey agar (Oxoid, Ltd., UK) by streaking method using a standard calibrated wire loop having capacity of containing 0.001 ml of urine. The inoculated plates were incubated aerobically at 37°C for 18–24 hours [13]. Reinoculation was done for four media with growth not prominent enough to allow for decision on the identity of bacteria, other 24 hours required for reinoculation. Primary isolation of bacteria was made based on their colony characteristics. For instance, *E. coli* were lactose-fermenting mucoid colonies on MacConkey agar while *Proteus* spp. produced striking swarming colonies on the blood agar plate. Then, further identification of bacterial organism to species level was carried out by taking an isolated colony from a subculture and inoculated onto different biochemical media. In short, Gram-negative bacteria were identified by conducting a series of biochemical tests such as Kligler's Iron Agar (KIA), Sulphur Indole Motility (SIM) media, citrate, oxidase, urease, and catalase and coagulase were used for identification of Gram-positive bacteria.

Then, the confirmed colony was counted from CLED media and multiplied by 1000 to determine the number of bacteria per ml (CFU) of the original urine specimen. A specimen was considered positive for ASB if a single organism was growing at a concentration of ≥10^5^ colony-forming units/ml. A single isolated bacterium was inoculated onto nutrient agar slant and stored in a refrigerator after 24 hours of incubation for the maintenance of isolated bacteria.

#### 2.3.4. Antimicrobial Susceptibility Testing

Antimicrobial susceptibility testing was performed using the disc diffusion method as described by the Clinical Laboratory Standards Institute [14]. In brief, the pure culture was transferred to a tube containing 5 ml sterile normal saline (0.85% NaCl) and mixed gently until it formed a homogeneous suspension. The turbidity of the suspension was adjusted to the optical density equivalent to 0.5 McFarland. A sterile cotton swab was then dipped into the suspension and the excess was removed by gentle rotation of the swab against the surface of the tube. The swab was distributed evenly over the entire surface of Mueller-Hinton agar (Oxoid, Ltd., UK).

The inoculated plates were left at room temperature to dry for 3 to 5 minutes. The antimicrobial discs (Oxoid, Ltd., UK) representative of the penicillin group (Amoxicillin 10 *μ*g, Augmentin 30 *μ*g, and Ampicillin 10 *μ*g), Cephalosporin (Ceftriaxone 30 *μ*g), aminoglycosides (Gentamycin 10 *μ*g), Maxolid (Erythromycin 15 *μ*g), Lincosamide (Clindamycin 2 *μ*g), Sulfonamide (Cotrimoxazole 25 *μ*g, and Tetracycline 10 *μ*g were placed on the inoculated plates and incubated at 37°C for 18–24 hours.

#### 2.3.5. Operational Definition

*(1) Asymptomatic*. means pregnant women who do not have the sign and symptom of UTI.

*(2) Antimicrobial Susceptibility Pattern*. is defined as *sensitive* when the inhibition zone of the isolated bacteria is greater than the inhibition zone set for the antimicrobial disk mentioned in the testing panel, *resistant* when the inhibition zone of isolated bacteria is less than the inhibition zone set for the respective antimicrobial disk used in the testing panel, and *intermediate* when the inhibition zone of isolated bacteria is less or equal to the inhibition zone set for the antimicrobial disk mentioned in the testing panel (Oxoid, Ltd., UK).

*(3) Multidrug Resistance (MDR)*. refers to bacteria showing resistance by two or more than different groups of antimicrobial agents in the testing panel.

*(4) Significant Bacteriuria*. refers to the presence of ≥10^5^ colony-forming units per milliliter in the urine sample.

### 2.4. Method of Data Analysis

Data were double entered into EpiData version 3.1, cleaned, and exported to Statistical Package for Social Science (SPSS) version 22. Descriptive statistics were performed for different variables. The results were presented in texts, tables, and graphs using summary measures such as percentages, standard deviation, and mean. The magnitude of asymptomatic bacteriuria was determined as proportion of positive urine culture to the total pregnant women participated in this study. Logistic regression was carried out to identify the associated factors with asymptomatic bacteriuria. Variables with *p* value <0.25 in bivariate analysis were considered as candidates for multivariable logistic regression to control confounder factors. The level of statistical significance was declared at *p* value <0.05.

### 2.5. Quality Control

A structured questionnaire initially prepared in English language was translated into Afan Oromo and Amharic by a language expert and translated back to English by another language expert. The questionnaire was pretested on 5% of the total sample size at the Jugal Hospital, Eastern Ethiopia, to ensure its validity. Completeness of each questionnaire was checked daily during data collection period.

The sterility of each medium was checked by incubating (3–5% of the batch) at 35–37°C overnight and looked for bacterial growth. The media which showed growth were discarded and replaced by a new sterile batch. The reference of American Type Culture Collection strains including *S. aureus* (ATCC 25923), *E. coli* (ATCC 25922), and *P. aeruginosa* (ATCC 27853) were used as a quality control throughout the study for culture and antimicrobial susceptibility test. Consistency of the entered data was validated by comparing the two separately entered data on EpiData version 3.1 (CDC/WHO, Atlanta, USA) by a data clerk having diploma in computer science. The frequencies of every variable were verified with the study population.

### 2.6. Ethical Consideration

The study protocol was reviewed and ethical clearance was obtained before starting the data collection process from the Institutional Health Research Ethics Review Committee (IHRERC) of the College of Health and Medical Sciences. Official letters of cooperation was submitted to Hiwot Fana Specialized University Hospital. The study participants were informed of their right to refuse or decline participation in the study at any time and that refusing to participate in the study will not affect them. Informed voluntary, written, and signed consent was obtained from all respondents before the study. The confidentiality of participants' information was assured by excluding names and identifiers in the questionnaire.

## 3. Results

### 3.1. Sociodemographic Characteristics of the Study Participants

In this study, a total of 281 asymptomatic pregnant women were included with a response rate of 99.3%. The age of the study participants ranges from 18 to 41 years with mean age ±standard deviations (SD) of 26.3 ± 5.7 years. Among the study participants, 91.8%, 44.8%, and 39.9% were married, urban dwellers, and with primary level education (1 to 8 grades), respectively. In addition to this, 64.4%, 34.2%, and 43.8% were house wife, primigravida, and at second trimester, respectively. Regarding participants' average monthly income, about 32% of the study participants had average monthly income less than 1000 Ethiopian birr (Table 1).

### 3.2. Clinical, Behavioral, and Pregnancy-Related Factors

Among the study participants; 53.7%, 81.1%, and 58.4% had second antenatal care (ANC) visit, performed sexual activities less than two times per week, and the habit of postcoital washing, respectively. In addition to this, 76.9%, 60.1%, 37.5%, and 25.6% of the study participants had no habit of postcoital urination, wiped their genitals after washing from front to back direction, and history of UTI and antibiotic use before two weeks, respectively. Among the study participants; 8.5%, 5.7%, 17.8%, and 68.7% had history of hypertension, diabetes, catheterization, and anemia, respectively (Table 2).

### 3.3. Asymptomatic Bacteriuria and Types of Bacterial Isolates

In this study, the overall prevalence of significant bacteriuria was 19.9% (95% CI: 16.4% to 24.6%). Six different bacterial isolates were identified. Gram-negative bacteria were the predominant isolates (60.7%), of which *E. coli* (44.6%) was the most predominant isolate followed by *Klebsiella* spp. (8.9%). Among Gram-positive bacteria, Coagulase-negative staphylococci*CoNS* were more frequently isolated (28.6%) followed by *S. aureus* (10.7%).

### 3.4. Factors Associated with Asymptomatic Bacteriuria

In bivariate analysis, variables such as history of UTI, catheterization, the age group of 18–26 years old, the average monthly income of the participants <1000 birr, habit of postcoital urination, direction of wiping after genital wash, and educational status were significant at *p* value <0.25 and candidates for multivariable analysis.

In multivariable analysis, catheterization, direction of wiping after genital wash, postcoital urination remained statistically significant at *p* value less than 0.05. Study participants who have experienced catheterization had higher odds of significant bacteriuria than those who did not experience catheterization (AOR = 3.214; 95% CI: 1.643, 6.284). The study participants who wipe their genitals after genital wash from back to front were 2.9 times more likely to develop ASB than those who wipe from front to back direction (AOR = 2.913; 95% CI: 1.595, 5.320). The study participants who do not have the habit of postcoital urination had higher odds than those who have the habit of postcoital urination (AOR = 2.96; 95% CI: 1.207, 7.266) (Table 3).

### 3.5. Gram-Positive Isolates

Most of the Gram-positive isolates were highly sensitive to Ceftriaxone (90.9%). *CoNS* showed higher sensitivity to Augmentin (75.0) and Ceftriaxone (87.5%), whereas they showed resistance to Clindamycin (68.7%) and Ampicillin (62.5%), while *S. aureus* showed sensitivity to Ceftriaxone (100%), Gentamycin (66.7%), and Augmentin (66.7%). Most of the Gram-positive isolates were highly resistant to Ampicillin (63.6%) and Clindamycin (71.4%).

### 3.6. Gram-Negative Isolates

Gram-negative bacteria isolates showed higher sensitivity to Ceftriaxone (88.2%), Gentamycin (67.5%), and Augmentin (64.7%), while showed resistance to Ampicillin (70.5%) and clindamycin (50.0%). From Gram-negative bacteria isolates, Pseudomonas spp. were 100% sensitivity to each of Gentamycin and Ceftriaxone, whereas they were 50% resistant to each of Ampicillin and Erythromycin. *E. coli* were sensitive to Ceftriaxone (92.0%), Gentamycin (68.0%), and Augmentin (60.0%), while they were resistant to Ampicillin (80.0%) (Table 4).

## 4. Discussion

In this study, the overall prevalence of ASB was 19.9%. Six different bacterial isolates were identified, of which *E.coli,* the most dominant isolate (44.6%), was followed by Coagulase-negative staphylococci (28.6) and *S. aureus* (10.7%). Direction of wiping after genital wash, catheterization, and postcoital urination increase the odds of asymptomatic bacteriuria.

This finding was consistent with previous studies from Adigrat, Ethiopia (21.2%) [5], and Ghana (17.5%) [14]. But it was relatively higher in other studies reported from Bahir Dar, Ethiopia (11.5%) [9], and countries such as India (13.2%) [15] and Nigeria (10.6%) [16]. On the contrary, this finding was relatively lower than the result reported from Saudi Arabia [17] and Egypt (29%) [18]. This difference in findings might be attributed to differences in sample size, geographical variation, and socioeconomic condition, awareness, and predisposing factors. There is a report, which indicates the prevalence of asymptomatic bacteriuria in pregnant women in the world which is known to vary from geographical region to region even within the same country [19].

Most of the isolated bacteria in this study were Gram negative (60.7%) which agrees with the study report from Adama, Ethiopia (72.6%) [10], and Kenya (55.3%) [19]. This finding is supported by the fact that Gram-negative bacteria have a unique structure which assists in attachment to the uroepithelium and prevents pathogens from being washed away by urine and the same characteristics allow for growth and tissue invasion resulting in invasive infection and pyelonephritis during pregnancy [1].

*Escherichia coli* was the most dominant isolate (44.6%) in this study. This finding was comparable with the study conducted in Ethiopia: Adigrat (34.6%) [5], Adama (37.3%) [10], and other parts of the world, such as India (57.7%) [1].

In this study, participants who wipe genitals after wash from back to front direction were more likely to develop ASB. This finding agrees with the finding reported from Cairo, Egypt [20] and Ismailia city of Egypt [18]. This is due to that bacteria from intestine might be transferred to female genital through wiping.

In the current study, participants who have experienced catheterization were three-fold more likely to develop ASB. This finding is similar to a study reported in Baghdad [4]. Catheter-associated ASB occurs when bacteria in a catheter bypass the body's defense mechanisms and enter the bladder [19]. However, the study conducted in Bahirdar, Ethiopia, [9] showed there was no significant association between catheterization and prevalence of ASB. The observed variation might be due to the variation in the catheterization technique/sterility and sample size difference.

In the current finding, study participants who have no habit of postcoital urination were two-fold more likely to develop ASB than their counterpart. Another study conducted in Cairo, Egypt, reported that the pregnant women who do not urinate after coitus were eight-fold more likely to develop ASB than those who have the habit of postcoital urination [21]. This might be because postcoital urination cleans the urethra from the bacteria entered during sexual intercourse.

In this study, Gram-positive isolates (*CoNS* and *S. aureus*) showed sensitivity to Ceftriaxone (90.5%), Augmentin (71.4%), and Gentamycin (52.4%) while they showed resistance to Ampicillin (63.6%) and Clindamycin (71.4%). On the contrary, the study conducted in Dessie Hospital, Ethiopia, showed that Gram-positive isolates showed sensitivity to Ceftriaxone (100%) and Clindamycin (62.2%) while they showed resistance to Tetracycline (62.2%) and Erythromycin (56.7%) [22].

Prescription of antibiotics without laboratory guidance as well as over-the-counter sales of antibiotics without prescription are a probable factor for increased bacterial resistance to antimicrobial agents [10, 22]. For instance, Ampicillin and other antimicrobials are freely available in local pharmacies, and people could purchase without prescription and use them with less adherence in Ethiopia. In general, the importance of screening and treatment of ASB include possible benefits such as reductions in incidence of maternal mortality, maternal sepsis, pyelonephritis, perinatal mortality (≥20 weeks of gestation), spontaneous abortion (<20 weeks of gestation), neonatal sepsis, preterm delivery (<37 weeks of gestation), and low birth weight (≤2500 g) [11, 12].

## 5. Conclusion

The overall prevalence of ASB among pregnant women in the present study was higher than previous studies conducted in Ethiopia. The current study revealed that postcoital urination, catheterization, and direction of wiping after genital wash were the factors that aggravated the occurrence of ASB in pregnant women. The most frequently identified isolate was *E. coli* (44.6%). Most of the bacterial isolates in this study were sensitive to Ceftriaxone and Augmentin while they were resistant to Ampicillin and Clindamycin. Of the total isolated bacteria, 54% of Gram-positive and 61.8% of Gram-negative bacteria showed resistance to two or more antimicrobials. Based on the above mentioned finding, health education should be provided for pregnant women on the identified ASB predisposing factors. Ceftriaxone, Augmentin, and Gentamycin can be used for the treatment of ASB among pregnant women, while Ampicillin and Clindamycin might not be used for ASB treatment in the study area. Finally, microbiological examinations for the isolation and culture identification of the bacteria causing ASB should be performed and selection of the appropriate antibiotic should be based on the drug susceptibility pattern of isolated bacteria.

## Figures and Tables

**Table 1:** Sociodemographic characteristics of pregnant women attending at Hiwot Fana Specialized University Hospital, Harar, Eastern Ethiopia, 2019 (*n* = 281).

Characteristics	Frequency number (%)
*Age group*	
18–26	157 (55.9)
27–34	107 (38.1)
35–44	17 (6.0)

*Marital status*	
Married	258 (91.8)
Divorced	20 (7.1)
Single	1 (0.4)
Widowed	2 (0.7)

*Residence*	
Urban	126 (44.8)
Rural	155 (55.2)

*Monthly income*	
<1000	90 (32)
1000–1999	50 (17.8)
2000–2999	72 (25.6)
>2999	69 (24.6)

*Religion*	
Orthodox	56 (19.9)
Protestant	32 (11.4)
Muslim	190 (67.6)
Others	3 (1.1)

*Educational status*	
Do not read and write	78 (27.8)
Primary (1–8 grade) school	112 (39.9)
Secondary (9–12 grade) school	45 (16.0)
College and above	46 (16.4)

*Occupation*	
Student	8 (2.8)
House wife	181 (64.4)
Merchant	41 (14.6)
Civil servant	49 (17.4)
Others	2 (0.7)

**Table 2:** Pregnancy, Clinical condition, and behavior-related factors of pregnant women attending at Hiwot Fana Specialized University Hospital, Harar, Eastern Ethiopia, 2019 (*n* = 281).

Characteristics	Frequency (%)
*Gravida*	
Nulligravida	69 (24.6)
Primigravida	96 (34.2)
Multigravida	58 (20.6)
Grand multigravida	58 (20.6)

*Gestational period*	
First trimester	43 (15.3)
Second trimester	123 (43.8)
Third trimester	115 (41.0)

*ANC follow-up*	
First visit	65 (23.1)
Second visit	151 (53.7)
Third visit	35 (12.5)
Fourth visit	30 (10.7)

*History of UTI*	
Yes	105 (37.5)
No	176 (62.7)

*History of drug use*	
Yes	72 (25.6)
No	209 (74.4)

*Hypertension*	
Yes	24 (8.5)
No	257 (91.5)

*Diabetes*	
Yes	16 (5.7)
No	265 (94.3)

*Hemoglobin level*	
Anemic	193 (68.7)
Nonanemic	88 (31.3)

*History of catheterization*	
Yes	50 (17.8)
No	231 (82.2)

*Postcoital urination*	
Yes	65 (23.1)
No	216 (76.9)

*Postcoital washing*	
Yes	165 (58.4)
No	116 (41.3)

*Direction of wiping after wash*	
Backward	169 (60.1)
Forward	112 (39.9)

*Sexual activities per week*	
<2 times	228 (81.1)
>2 times	53 (18.9)

**Table 3:** Pregnancy-related and personal factors associated with ASB among pregnant women participated in the study at Hiwot Fana Specialized University Hospital, Harar, Eastern Ethiopia, 2019 (*n* = 281).

Characteristics	Significant bacteriuria no. (%)	Nonsignificant bacteriuria no. (%)	Crude odds ratio (95% CI)	*p* value	Adjusted odds ratio (95% CI)
*Gravida*					
Nulligravida	14 (25.0)	55 (24.2)	1		
Primigravida	18 (32.1)	78 (34.8)	1.025 (0.432, 2.434)	0.805	
Multigravida	12 (21.4)	46 (20.4)	1.130 (0.500, 2.557)	0.956	
Grand multigravida	12 (21.4)	46 (20.4)	1.000 (0.407, 2.456)	0.956	

*Gestational period*					
First trimester	10 (17.9)	36 (15.9)	1		
Second trimester	23 (41.1)	102 (44.9)	1.186 (0.510, 2.754)	0.520	
Third trimester	23 (41.1)	89 (39.2)	1.99 (0.661, 1.506)	0.692	

*Hemoglobin level*					
Anemic	40 (71.4)	153 (68.0)	1.185 (0.623, 2.255)	0.621	
Nonanemic	16 (28.6)	72 (32.0)	1		

*Sexual activities/week*					
0–2	43 (76.8)	185 (82.2)	1		
≥2	13 (23.2)	40 (17.8)	0.715 (0.352, 1.452)	0.354	

*Hypertension*					
Yes	7(12.5)	17 (7.5)	0.929 (0.296, 2.915)	0.899	
No	49 (87.5)	208 (92.4)	1		

*Diabetes*					
Yes	6 (10.7)	10 (6.3)	1.187 (0.387, 3.640)	0.764	
No	50 (89.3)	215 (93.7)	1		

*ANC follow-up*					
1st visit	16 (28.6)	49 (21.8)	1		
4th visit	6 (10.7)	24 (10.7)	0.766 (0.266, 2.205)	0.744	

*Postcoital washing*					
No	90 (51.8)	29 (39.6)	0.642 (0.357, 1.157)	0.24	1.277 (0.666, 2.447)
Yes	27 (48.2)	137 (60.4)	1	1	

*History of antimicrobial use*					
Yes	15 (26.8)	57 (25.1)	0.927 (0.478, 1.800)	0.824	
No	41 (73.2)	168 (74.9)	1		

*Catheterization*					
Yes	19 (38)	31 (62)	3.214 (1.643, 6.284)	0.001^*∗*^	3.122 (1.378, 7.072)
No	37 (16.1)	194 (83.9)	1	1	

*History of UTI*					
Yes	29 (51.8)	76 (33.8)	1		
No	27 (37.5)	149 (66.2)	0.478 [0.26, 0.864]	0.181^*∗*^	1.424 (0.719, 2.820)

*Postcoital urination*					
Yes	6 (10.7)	59 (26.0)	1		
No	50 (89.3)	168 (74.0)	2.962 (1.207, 7.266)	0.018^*∗*^	3.160 (1.173, 6.514)^*∗∗*^

*Direction of wiping after genital wash*					
Forward	34 (60.7)	79 (34.8)	2.913 (1.595, 5.320)	0.001^*∗*^	2.853 (1.255, 4.320)^*∗∗*^
Backward	22(39.3)	148 (65.2)	1		

CI, confidence interval; ^*∗*^*p* < 0.25; ^*∗∗*^*p* value <0.05; OR: odds ratio.

**Table 4:** Antimicrobial susceptibility pattern of Gram-negative bacteria isolated from urine culture of pregnant women with asymptomatic bacteriuria at Hiwot Fana Specialized University Hospital, Harar, Eastern Ethiopia, 2019.

Isolates	Number	Pattern	AMP	AUG	A	CN	COT	CD	T	CRO	E
*E. coli*	25		*N* (%)	*N* (%)	*N* (%)	*N* (%)	*N* (%)	*N* (%)	*N* (%)	*N* (%)	*N* (%)
		S	—	15 (60.0)	13 (52.0)	17 (68.0)	14 (56.0)	9 (36.0)	10 (40.0)	23 (92.0)	13 (52.0)
		I	5 (20.0)	4 (16.0)	3 (12.0)	4 (16.0)	3 (12.0)	4 (16.0)	5 (20.0)	1 (4.0)	4 (16.0)
		R	20 (80.0)	6 (24.0)	9 (36.0)	4 (16.0)	8 (32.0)	12 (48.0)	10 (40.0)	1 (4.0)	7 (28.0)

*Klebsiella* spp.	5										
		S	1 (20)	4 (80)	2 (40)	3 (60)	3 (60)	2 (40)	2 (40)	3 (60)	—
		I	3 (60)	—	—	2 (40)	2 (40)	—	1 (20)	2 (40)	1 (20)
		R	1 (20)	1 (20)	3 (60)	—	—	3 (60)	2 (40)	—	4 (80)

*Pseudomonas* spp.	2	S	1 (50)	1 (50)	1 (50)	2 (100)	—	1 (50)	1 (50)	2 (100)	—
		I	—	—	—	—	2 (100)	1 (50)	—	—	1 (50)
		R	1 (50)	1 (50)	1 (50)	—	—	—	1 (50)	—	1 (50)

*Proteus* spp.	2										
		S	—	2 (100)	2 (100)	1 (50)	—	—	—	2 (100)	1 (50)
		I	—	—	—	—	—	—	—	—	—
		R	2 (100)			1 (50)	2 (100)	2 (100)	2 (100)	—	1 (50)

Total	34										
		S	2 (5.8)	22 (64.7)	18 (52.9)	23 (67.5)	17 (50.0)	12 (35.3)	13 (38.2)	30 (88.2)	15 (44.0)
		I	8 (23.5)	4 (11.8)	3 (8.8)	6 (17.8)	7 (20.5)	5 (14.7)	6 (17.8)	3 (8.8)	6 (17.8)
		R	24 (70.5)	8 (23.5)	13 (38.2)	5 (14.7)	10 (29.4)	17 (50.0)	15 (44.0)	1 (3.0)	13 (38.2)

AMP, Ampicillin; CRO, Ceftriaxone; A, Amoxicillin; CN, Gentamicin; COT, Cotrimoxazole; AUG, Augmentin; ERY, Erythromycin; CD, Clindamycin; T, Tetracycline; *CoNS*, coagulase-negative staphylococci species; S, sensitive; I, intermediate; R, resistant.

## Data Availability

The dataset used/or analyzed during the current study are available from the author on reasonable request.
